# Clinical characteristics and sequelae of intrapartum hypertension – a retrospective cohort study

**DOI:** 10.1186/s12884-023-05386-y

**Published:** 2023-03-06

**Authors:** Theepika Rajkumar, Annemarie Hennessy, Yumna Ali, Angela Makris

**Affiliations:** 1 Western Sydney University, NSW Sydney, Australia; 2 Department of Medicine, Campbelltown Hospital, NSW 2560 Campbelltown, Australia; 3 Liverpool Hospital, NSW Sydney, Australia; 4 Department of Renal Medicine, Liverpool Hospital, NSW Sydney, Australia; 5 University of New South Wales, NSW Sydney, Australia

**Keywords:** Intrapartum hypertension, Hypertensive disorders of pregnancy, Gestational hypertension, Maternal morbidity, Fetal morbidity, Delivery complications

## Abstract

**Background:**

In a significant proportion of pregnant women, elevated blood pressure may first present during the intrapartum period. This phenomenon, intrapartum hypertension, is often overlooked as blood pressure during delivery is attributed to labour pain, analgesic agents and haemodynamic changes. Thus the true prevalence and clinical significance of intrapartum hypertension remains unknown. This study sought to define the prevalence of intrapartum hypertension in previously normotensive women, identify associated clinical characteristics, and its impact on maternal and fetal outcomes.

**Methods:**

In this single-center retrospective cohort study, all available partograms were reviewed over a 1-month period at an outer metropolitan hospital in Sydney (Campbelltown Hospital). Women with diagnosed hypertensive disorders of pregnancy during the incident pregnancy were excluded. A total of 229 deliveries were included in the final analysis. Intrapatum hypertension (IH) was defined as two or more systolic blood pressure (SBP)⩾140 mmHg or diastolic blood pressure (DBP)⩾90 mmHg during the intrapartum. Demographic data at the time of the first antenatal visit for the incident pregnancy as well as final maternal outcomes (intrapartum and post-partum) and fetal outcomes were collected. Statistical analyses were carried out using SPSSv27 with adjustments for baseline variables.

**Results:**

Amongst 229 deliveries, 32 women (14%) had intrapartum hypertension. Older maternal age (*p* = 0.02), higher body mass index (*p* < 0.01) and higher diastolic blood pressure at the first antenatal visit (*p* = 0.03) were associated with intrapartum hypertension. A longer second stage of labour (*p* = 0.03), intrapartum non-steroidal anti-inflammatory medications (*p* < 0.01) and epidural anaesthesia (*p* = 0.03) were associated with intrapartum hypertension, while IV syntocin for labour induction was not. Women with intrapartum hypertension had a longer inpatient admission following delivery (*p* < 0.01), and elevated postpartum BP (*p* = 0.02) with discharge on antihypertensive medications (*p* < 0.01). Intrapartum hypertension was not associated with poor fetal outcomes, though subgroup analyses showed that women who had at least a single elevated blood pressure reading during the intrapartum experienced poorer fetal outcomes.

**Conclusion:**

In previously normotensive women, 14% developed intrapartum hypertension during delivery. This was associated with postpartum hypertension, longer maternal admission and discharge with antihypertensive medications. There was no difference in fetal outcomes.

**Supplementary Information:**

The online version contains supplementary material available at 10.1186/s12884-023-05386-y.

## Background

Hypertensive disorders of pregnancy are an important cause of maternal and neonatal morbidity worldwide, and there has been an increasing focus during antenatal care to initiate appropriate treatment and reduce poor perinatal outcomes [1]. In women without pre-existing risk factors, the onset of a hypertensive disorder of pregnancy (HDP) is most commonly in the third trimester [1]. However, for a significant proportion of women, this may first present during labour. There is little data on the clinical significance of intrapartum hypertension (IH). Intrapartum hypertension is often overlooked in clinical practice as blood pressure measurements during labour may be affected by pain, analgesic agents and haemodynamic changes. The lack of consensus about the diagnostic criteria for labour-onset hypertension further compounds the problem and competing priorities make management decisions difficult. In a low-risk midwifery-led obstetric population, a 2% rate of elevated blood pressure during delivery was reported [2], while Ohno et al [3] found in a more heterogenous population, that 24% of pregnant women who remained normotensive throughout pregnancy developed hypertension during labour. Risk factors that have been identified for IH include advancing maternal age, obesity and nulliparity [2–4].

Despite the incongruency in reported prevalence, the limited existing literature suggests an association with poor outcomes. Lao et al. [5] reported that in 53% of cases of eclampsia during labour, the hypertension first presented only after labour onset. Similarly, Ohno et al [3] showed that hypertension first presented after labour onset in 55% (41/75 cases) of patients who developed eclampsia and 56% (5/9 cases) of those who suffered strokes during labour.

Thus, the objective of this study was to define the prevalence of IH in women who were previously normotensive throughout their pregnancy. The study also sought to identify the clinical characteristics associated with IH, and the consequences for maternal and fetal outcomes.

## Methods

A single-center retrospective cohort study was conducted. All available electronic and paper partograms were reviewed over a 1-month (October 2019) period at an outer metropolitan hospital in Sydney. Campbelltown Hospital is a teaching hospital in South Western Sydney with over 3400 births annually. Women more than 32 weeks gestation are able to deliver at Campbelltown Hospital as it has a special care nursery but no neonatal intensive care.

All women with a partogram who delivered during the study period were included. Partograms without any blood pressure readings recorded were excluded. Women with a diagnosis of a hypertensive disorder of pregnancy (preeclampsia, chronic hypertension, gestational hypertension or superimposed preeclampsia on chronic hypertension [6]) prior to their delivery admission were excluded from the final analysis, as the aim was to focus on new onset IH in previously normotensive women.

Labour partograms were reviewed and IH was defined as two or more either systolic blood pressure (SBP) measurement ⩾140 mmHg or diastolic blood pressure (DBP) ⩾90 mmHg during the intrapartum period, at least 30 minutes apart. For labouring women without a pre-existing diagnosis of HDP, blood pressure is checked every 2 h using an automated blood pressure device. Hospital policy encourages women to adopt positions that enhance labour and uteroplacental perfusion, and women are not repositioned to check blood pressure. For women undergoing a caesarean section, continuous non-invasive blood pressure monitoring occurs intraoperatively using an automated blood pressure device, in the supine position.

A subgroup analysis was also undertaken for women with a single SBP measurement ⩾140 mmHg or DBP measurement ⩾90 mmHg during labour. The length of each stage of labour, total number of readings measured, total number of elevated readings and medication charts were reviewed. The post partum course was assessed for episodes of hypertension, medications commenced, length of stay and adverse maternal or fetal outcomes (maternal outcomes: mortality, stroke, acute coronary event, eclampsia, acute kidney injury, raised liver enzymes, pulmonary oedema, placental abruption, ICU admission or hospital readmission within 6 weeks of delivery; fetal outcomess: mortality, admission to the special care nursery or neonatal intensive care unit, stillbirth, preterm at delivery, seizures, intrauterine growth restriction or small-for-gestational age [7]). A maternal and fetal composite outcome score was defined based on these outcomes. Fetal weight centiles were calculated using the Intergrowth calculator (https://intergrowth21.tghn.org).

Electronic documentation were reviewed for a total of 6 weeks follow-up to assess for re-admissions due to uncontrolled or symptomatic hypertension, in addition to the hypertensive complications listed above. Readmissions for other causes were not included unless they had concomitant hypertensive complications, or were noted to have elevated blood pressure during their admission requiring medication changes.

Ethics for this study was approved by the South Western Sydney Local Health District Research Ethics committee (2020/ETH00824). Statistical analyses were carried out using SPSSv27 with adjustments for baseline variables. Data that is normally distributed was analysed with parametric tests and non-normally distributed data was analysed with non-parametric tests. Significance was set at 0.05.

## Results

**Fig. 1:**
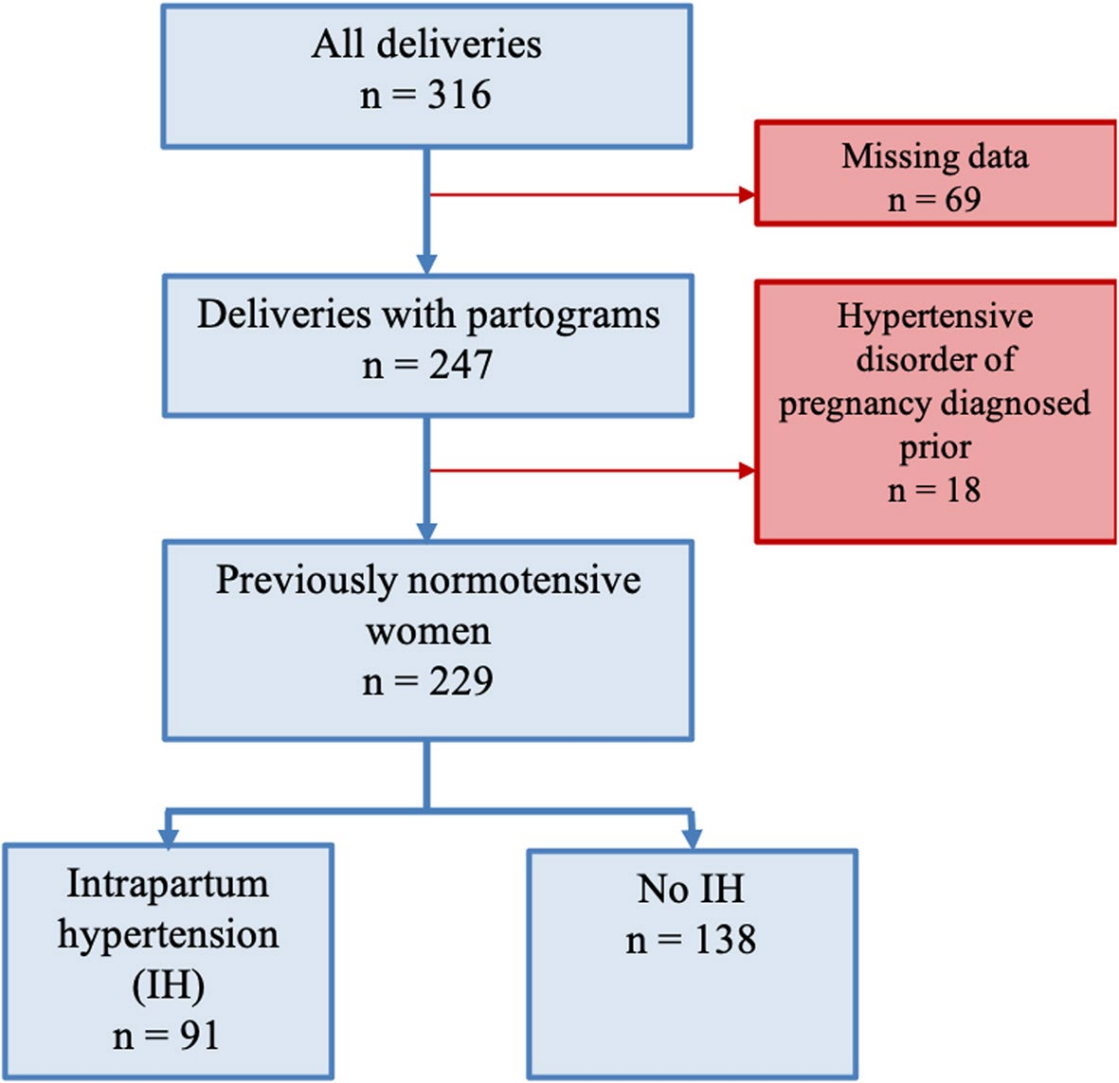
Flow of patients in this study

During October 2019, there were 316 births at Campbelltown Hospital, with 300 labour partograms available for review. Fifty-three partograms (18%) had no blood pressure readings documented during the intrapartum period. A further 18 women were excluded due to having a diagnosis of a hypertensive disorder of pregnancy. The flow of patients is outlined in Fig. 1.

**Fig. 2:**
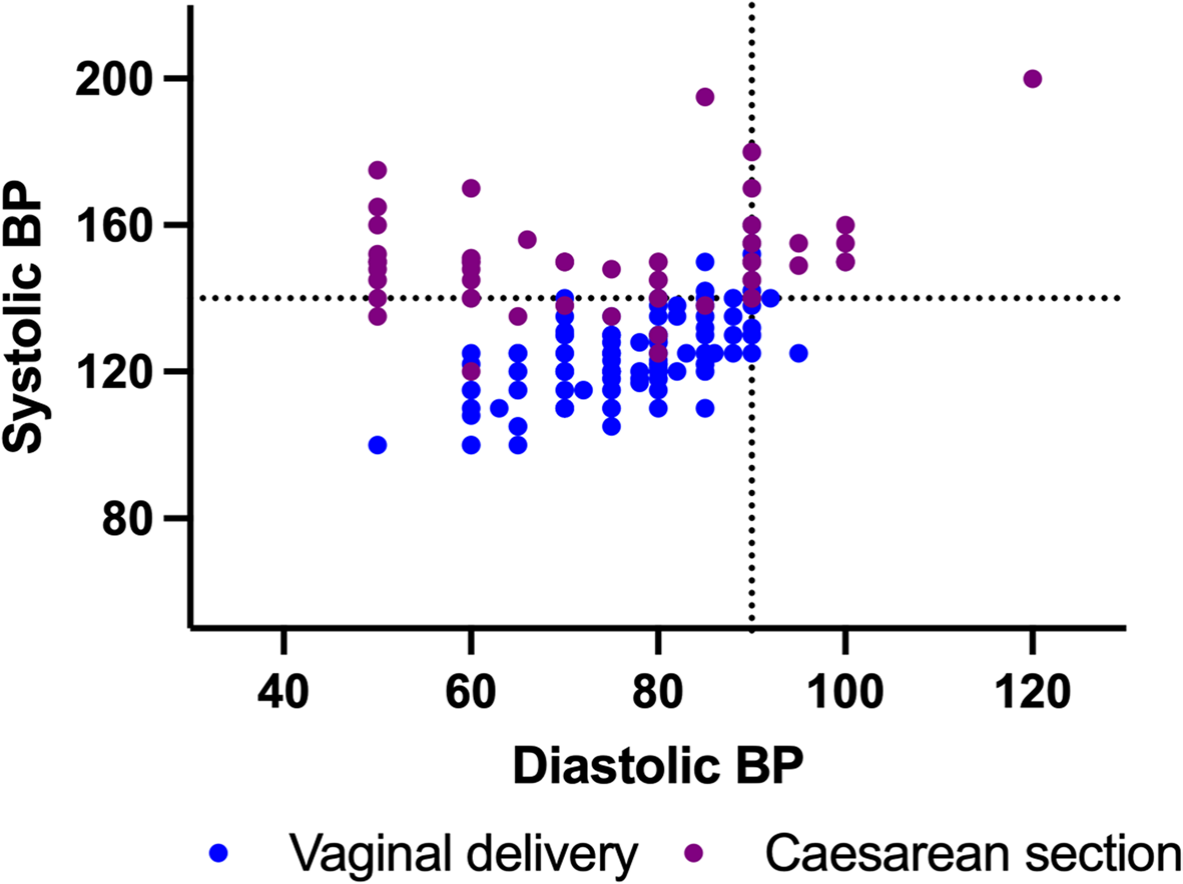
Scatter plot of all the blood pressure readings

The median (±IQR) number of blood pressure readings per patient was 4 (±21), with caesarean sections recording more blood pressure readings than a vaginal delivery (31 ± 16 and 2 ± 4 respectively; *p* < 0.001). Figure 2 outlines the distribution of blood pressure readings collected in this cohort.

Amongst 229 women included in the final analysis, two or more elevated intrapartum blood pressure readings were recorded in 32 women (14%), and this was associated with higher maternal age (*p* = 0.02), higher body mass index (*p* < 0.001) and higher DBP at the first antenatal visit (*p* = 0.03) (Table 1). All other baseline demographics were similar between the two groups.

**Table 1:** Baseline demographics

	Total (*n* = 229)	⩾2 elevated BP readings (*n* = 32)	< 2 elevated BP readings (*n* = 197)	*p*-value
Age (mean ± SD)	30.1 ± 5.3	32 ± 4.8	29.74 ± 5.3	0.02
Gravidity (median ± IQR)	2 ± 2	2 ± 2	2 ± 2	NS
Parity (median ± IQR)	1 ± 2	1 ± 1	1 ± 2	NS
BMI (median ± IQR)	26.2 ± 8.7	31.6 ± 11.3	25.50 ± 8.2	< 0.001
Gestation at first antenatal visit (median ± IQR)	16 ± 6	14.5 ± 6.5	16 ± 6	NS
First antenatal visit SBP mmHg (median ± IQR)	100 ± 10	106.5 ± 16.5	100 ± 10	NS
First antenatal visit DBP mmHg (median ± IQR)	60 ± 10	65 ± 10	60 ± 8	0.03
Gestation at delivery (median ± IQR)	39 ± 2	39 ± 2	39 ± 2	NS

*IH* intrapartum hypertension, *SD* standard deviation, *IQR* interquartile range, *BMI* body mass index, *SBP* systolic blood pressure, *DBP* diastolic blood pressure, *PET* preeclampsia, *HTN* hypertension, *NS* non-significant, *SROM* spontaneous rupture of membranes, *IOL* induction of labour, *LSCS* lower segment caesarean section, *GDM* gestational diabetes mellitus, *PROM* preterm rupture of membranes, *AFI* amniotic fluid index

Thirty-one women (13.5%) had an intrapartum systolic blood pressure reading ⩾140 mmHg, 7 (3%) had intrapartum systolic blood pressure reading ⩾160 mmHg, and 16 (7%) had an intrapartum diastolic blood pressure reading ⩾90 mmHg.

Table 2 outlines characteristics of delivery for the IH and no IH groups. Women with ⩾2 or more elevated blood pressure readings during the intrapartum were more likely to undergo an emergency caesarean section (*p* < 0.01) and have a longer second stage of labour (*p* = 0.03).

**Table 2:** Intrapartum characteristics of IH and no IH groups

	Total (*n* = 229)	⩾2 elevated BP readings (*n* = 32)	< 2 elevated BP readings (*n* = 197)	*p*-value	Unadjusted odds ratio (95%CI)
Spontaneous or induced labour onset	188	16 (8.5%)	172 (91.5%)	< 0.001	0.15 (0.07–0.33)
Normal vaginal delivery	157	7 (4.5%)	150 (95.5%)		0.09 (0.04–0.22)
Emergency caesarean section	31	9 (29%)	22 (71%)		3.1 (1.3–7.6)
No labour	41	16 (39%)	25 (61%)		
Duration of first stage (median ± IQR)	3:30 (± 3:37)	3:30 (± 3:00)	3:30 (± 3:40)	NS	
Duration of second stage (median ± IQR)	0:28 (± 1:34)	02:08 (± 0:51)	00:24 (± 1:15)	0.03	
Intravenous syntocin, *n* (%)	86	13 (15.1%)	73 (84.9%)	NS	
Epidural, *n* (%)	70	15 (21.4%)	55 (78.6%)	0.03	2.28 (1.06–4.88)
Intrapartum non-steroidal anti-inflammatory drugs, *n* (%)	45	17 (37.7%)	28 (62.2%)	< 0.001	6.8 (3.07–15.24)
Instrumental delivery, *n* (%)	34	5 (14.7%)	29 (85.3%)	NS	
Forceps	6	1 (16.7%)	5 (83.3%)		
Vacuum	28	4 (14.3%)	24 (85.7%)		
Emergency caesarean section, *n* (%)	31	9 (29%)	22 (71%)	0.009	3.1 (1.3–7.6)
Third- or fourth-degree perineal tear, *n* (%)	5	0 (0%)	5 (100%)	< 0.01	2.39 (1.22–4.66)

Administration of intrapartum non-steroidal anti-inflammatory medications (NSAIDs) (*p* < 0.01) and epidural anaesthesia (*p* = 0.03) were associated with IH, however administration of intravenous syntocin for labour induction was not associated. Seven women were treated with antihypertensive agents during the intrapartum period (*n* = 5 in the IH group, *n* = 2 in the no IH group). Intravenous antihypertensive agents were required in 3 women (*n* = 2 in the IH group, *n* = 1 in the no IH group). The median (± IQR) number of antihypertensive doses received was 2 (± 2).

Women with IH were more likely to have elevated blood pressure postpartum (*p* = 0.02), require a longer hospital admission following delivery (*p* < 0.01) and be discharged on regular antihypertensive medications (*p* < 0.01). No woman in either group suffered a stroke, seizure, acute coronary event, pulmonary oedema or other end-organ hypertensive complications. Three women were readmitted in the first 6 weeks post partum with uncontrolled hypertension, and none of these women had IH. Median (±IQR) time after discharge to readmission was 6 (±4) days, with the median (±IQR) readmission length 2 (±2) days.

Fetal outcomes were not significantly different between the two groups (Table 2).

Regression analysis was conducted to examine the relationship between IH and various potential predictors. Having 2 or more raised blood pressure readings during the intrapartum period was predictive of postpartum hypertension (OR 12.69; CI 1.36–118.22, *p* = 0.03) after controlling for the other variables in the model.

### Subgroup analysis of women with at least a single elevated (SBP⩾140 or DBP⩾90) reading in the intrapartum

Ninety-one women (39.7%) had at least a single raised blood pressure in the intrapartum period (SBP⩾140 or DBP⩾90), and this was associated with a higher body mass index (*p* = 0.02) and higher SBP at the first antenatal visit (*p* = 0.04).

There was an association between raised blood pressure during the intrapartum, and a longer second stage of labour (*p* = 0.03), as well as the need for an emergency caesarean section (*p* < 0.01). Administration of intrapartum non-steroidal anti-inflammatory medications (NSAIDs) (*p* < 0.01) and epidural anaesthesia (*p* < 0.01) were associated with IH.

Fetal outcomes and maternal post-partum outcomes differed between the two groups (Table 3). Intrapartum hypertension was also associated with an APGAR score < 9 at 1 minute and 5 minutes (*p* = 0.03 and *p* = 0.02 respectively), birthweight <10th centile (*p* < 0.01) and the need for high level neonatal care (*p* = 0.02). The group with at least a single raised blood pressure reading during the intrapartum was more likely to have a fetal composite outcome (*p* = 0.03).

**Table 3:** Fetal and maternal post partum outcomes in the IH and no IH groups

	Total (*n* = 229)	⩾2 elevated BP readings (*n* = 32)	< 2 elevated BP readings (*n* = 197)	*p*-value	Unadjusted odds ratio (95%CI)
**Fetal outcomes**					
Male infant, *n* (%)	118	18 (15.3%)	100 (84.7%)	NS	
1 minute APGAR score < 9, *n* (%)	21	5 (23.8%)	16 (76.2%)	NS	
5 minute APGAR score < 9, *n* (%)	9	3 (33.3%)	6 (66.7%)	NS	
Fetal weight (median ± IQR)	3468 (± 746)	3420 (± 904)	3473 (± 695)	NS	
Small-for-gestational age at delivery (<10th centile)	13	3 (23.1%)	10 (76.9%)	NS	
Intrauterine growth restriction at delivery (<5th centile)	5	1 (20%)	4 (80%)	NS	
Preterm delivery (< 37 weeks), *n* (%)	10	1 (10%)	9 (90%)	NS	
Admission to special care nursery/neonatal intensive care unit, *n* (%)	35	8 (22.9%)	27 (77.1%)	NS	
Fetal composite outcome^a^	40	9 (22.5%)	31 (77.5%)	NS	
**Maternal outcomes**					
Maternal ICU admission, *n* (%)	0	0 (0%)	0 (0%)	–	
Length of stay following delivery (median ± IQR)	2 (± 2)	3 (± 1)	2 (± 1)	< 0.01	
Length of stay > 3 days, *n* (%)	17	4 (23.5%)	13 (76.5%)	NS	
Postpartum hypertension, *n* (%)	19	6 (31.6%)	13 (68.4%)	0.02	3.27 (1.14–9.34)
Required IV antihypertensive agents	0	0 (0%)	0 (0%)	–	
Discharged with antihypertensive medications	4	4 (100%)	0 (0%)	< 0.01	1.14 (1.00–1.30)
Readmission due to hypertension, *n* (%)	3	0 (0%)	3 (100%)	NS	
Maternal composite outcome^b^	5	2 (40%)	3 (60%)	NS	

*IH* intrapartum hypertension, *SD* standard deviation, *IQR* interquartile range, *BMI* body mass index, *SBP* systolic blood pressure, *DBP* diastolic blood pressure, *PET* preeclampsia, *HTN* hypertension, *NS* non-significant, *SROM* spontaneous rupture of membranes, *IOL* induction of labour, *LSCS* lower segment caesarean section, *GDM* gestational diabetes mellitus, *PROM* preterm rupture of membranes, *AFI* amniotic fluid index, *APGAR* Appearance, Pulse, Grimace, Activity, and Respiration, *ICU* intensive care unit, *IV* intravenous

^a^Fetal composite outcome was defined as the composite of admission to the special care nursery or neonatal intensive care unit, stillbirth, preterm at delivery, neonatal mortality, neonatal seizures or intrauterine growth restriction (<5th centile)

^b^Maternal composite outcome was defined as the composite of maternal mortality, stroke, acute coronary event, eclampsia, acute kidney injury, raised liver enzymes, pulmonary oedema, placental abruption, ICU admission or readmission within 6 weeks of delivery

Women with at least a single raised intrapartum blood pressure reading also had a longer maternal inpatient admission following delivery (*p* < 0.01), and elevated postpartum blood pressure readings (*p* < 0.01) with discharge on regular antihypertensive medications (*p* = 0.01).

Regression analysis revealed that higher systolic blood pressure at the first antenatal visit (OR 1.05; CI 1.00–1.09, *p* = 0.04) was predictive of raised blood pressure during the intrapartum after controlling for the other variables of body mass index, intrapartum medications and duration of labour in the model.

### Subgroup analysis of excluded populations

Hypertensive disorders of pregnancy (HDP) had been diagnosed in 6% of women (*n* = 18) prior to delivery (gestational hypertension *n* = 10, preeclampsia *n* = 1, chronic hypertension *n* = 6, and superimposed preeclampsia on chronic hypertension *n* = 1), and 83% of this group had hypertension during labour. Compared to previously normotensive women, the HDP group were more likely to have significantly higher initial antenatal visit, intrapartum, postpartum and discharge systolic and diastolic blood pressures (*p* < 0.01). They were more likely to require intrapartum intravenous antihypertensive medications, as well as have antihypertensive medications on discharge (*p* < 0.01). This group had a significantly higher rate of preterm deliveries (*p* = 0.03), maternal ICU admissions (*p* < 0.01), maternal re-admissions (*p* < 0.01) and length of stay > 3 days (*p* < 0.01) compared to the previously normotensive population (Supplementary Table 2).

## Discussion

### Clinical characteristics associated with IH

The rate of IH over a 1-month period in this study cohort of previously normotensive women was 14%. The demographic factors associated with IH in this population included older maternal age, higher maternal BMI and a higher diastolic blood pressure at the first antenatal visit. These risk factors are consistent with previous work [3, 4], and suggests that traditional vascular risk factors increase risk of IH.

During the intrapartum a longer second stage of labour, intrapartum NSAIDs and epidural anaesthesia were associated with IH. It is unclear however, whether it is these medications themselves, or pain during delivery which account for this finding. Labour pain has been suggested to be responsible for increased cardiac output leading to increased blood pressure during labour [8], however, analgesic medications such as nonsteroidal anti-inflammatories also have known hypertensive effects. The haemodynamic effects of specific medications will always be difficult to elucidate in the context of confounding factors such as the physiology of delivery, blood loss, and the administration of other intrapartum medications, and it is likely that a combination of these factors contribute to this study finding.

This study also found that IH is more likely in women undergoing caesarean section, however, these women undergo close intra-operative monitoring and thus more likely to have elevated blood pressure recordings. Therefore, this finding should be interpreted with caution and future studies need to delineate whether this is a true association.

### Treatment of IH

One issue that was highlighted through this retrospective study was the low rates of treatment of severe IH. Twelve women (5.2%) developed systolic BP > 160 mmHg, though only seven were treated with antihypertensive agents, 3 of which required intravenous delivery. This is mirrored in other studies, where low rates of treatment of severe hypertension was common [3, 4, 9]. The reasons for this are varied, and include issues such as competing priorities, lack of knowledge about treatment parameters, fear of hypotension and reluctance to treat with intravenous medications [4]. Given the association of IH with poor maternal outcomes, it is clear that more diligence is required in instituting treatment once the condition is recognised. Prospective studies assessing the impact of early treatment of severe IH on maternal outcomes may be required to shift current practice.

### Impact of IH on maternal and fetal outcomes

In addition to defining the clinical characteristics of IH, this work sought to report on the consequences of IH for maternal and fetal outcomes. Two previous separate studies found that in more than half of eclampsia cases and intrapartum strokes, hypertension developed for the first-time during labour [3, 5]. In this study, though the maternal composite outcome was not different between groups, the findings confirm that IH is not benign for the mother. Intrapartum hypertension was associated with a longer maternal inpatient admission following delivery, and women were more likely to have postpartum hypertension and be discharged on regular antihypertensive medications. These findings are consistent with the hypothesis that IH more likely represents a late manifestation of a hypertensive disorder of pregnancy, rather than normal physiology of delivery [4].

This study is the first to assess fetal outcomes in the context of IH. There was no difference in fetal outcomes between the two groups. However, in the subgroup analysis of women with at least a single elevated BP reading, there was a higher rate of APGAR scores < 9, birthweight <10th centile, the need for high level neonatal care and a significant fetal composite outcome. The reason for this is unclear though may be in part explained by the higher rate of caesarean sections in this group, and larger studies are necessary to explore this association further.

Another possible explanation for the higher rate of fetal birthweight <10th centile is that these women had undiagnosed HDP.

### Limitations

There are several limitations to this study. Firstly, as a retrospective cohort study, the technique of measuring blood pressure that was utilised, nor the accuracy of recorded data within the medical record is not able to be validated. Secondly, this study had a small sample size, with no severe maternal events such as stroke or eclampsia occurring in this cohort.

Finally, there are sources of bias that arise from the disproportionate incidence of IH in women undergoing caesarean sections. There is potential selection bias as women may undergo a caesarean section because of IH. Additionally, women undergoing a caesarean section exclusively receive intrapartum NSAIDs, a practice undertaken by many anaesthetists locally to reduce post-operative pain [10], and tend to have a longer length of admission following surgery.

## Conclusions

Almost 14% of women without pre-existing hypertension, developed IH, while 3% developed severe hypertension (systolic BP ⩾160). Traditional vascular risk factors such as older maternal age, higher BMI and higher diastolic blood pressure at the first antenatal visit was associated with IH. Within this cohort, IH was associated with a longer maternal inpatient admission following delivery, elevated postpartum blood pressure readings, and discharge with regular antihypertensive medications. These findings suggest that IH may represent a late manifestation of a hypertensive disorder of pregnancy, rather than normal physiology of delivery.

## Supplementary Information

**Additional file 1: Supplementary Table 1.** Baseline demographics, intrapartum details and maternofetal outcomes for women two or more elevated blood pressure readings. **Supplementary Table 2.** Baseline demographics, intrapartum details and maternofetal outcomes for women with and without HDP.

## Data Availability

The datasets used and/or analysed during the current study are available from the corresponding author on reasonable request.

## Abbreviations

AFI: Amniotic fluid index
APGAR: Appearance, Pulse, Grimace, Activity, and Respiration
BMI: Body mass index
BP: Blood pressure
DBP: Diastolic blood pressure
GDM: Gestational diabetes mellitus
HDP: Hypertensive disorders of pregnancy
HTN: Hypertension
ICU: Intensive care unit
IH: Intrapartum hypertension
IOL: Induction of labour
IQR: Interquartile range
IV: Intravenous
LSCS: Lower segment caesarean section NS Non-significant
NSAID: Non-steroidal anti-inflammatory medications
PET: Preeclampsia
PROM: Preterm rupture of membranes
SBP: Systolic blood pressure
SD: Standard Deviation
SROM: Spontaneous rupture of membranes
