# Supplementary material for: Clinical characteristics and sequelae of intrapartum hypertension – a retrospective cohort study
Source: BMC Pregnancy Childbirth. 2023 Mar 6;23:146. doi: 10.1186/s12884-023-05386-y (PMC9987065; doi:10.1186/s12884-023-05386-y)
Supplement: Supplementary file 1 — Additional file 1: Supplementary Table 1. Baseline demographics, intrapartum details and maternofetal outcomes for women two or more elevated blood pressure readings. Supplementary Table 2. Baseline demographics, intrapartum details and maternofetal outcomes for women with and without HDP. [file 12884_2023_5386_MOESM1_ESM.pdf]

Supplementary material

Supplementary Table 1: Baseline demographics, intrapartum details and maternofetal outcomes for women two or more elevated blood pressure readings

	Total (n=229)	At least a single elevated blood pressure reading (n=91)	No elevated blood pressure readings (n=138)	p-value
Age (years; mean ± SD)	30.1 ± 5.3	30.4 ± 4.7	29.86 ± 5.6	NS
Gravidity (median ± IQR)	2 ± 2	2 ± 2	2 ± 3	NS
Parity (median ± IQR)	1 ± 2	0 ± 1	1 ± 2	NS
Booking BMI (median ± IQR)	26.2 ± 8.7	27.5 ± 10.8	25.50 ± 7.6	0.02
Gestation at booking (weeks: median ± IQR)	16 ± 6	15 ± 5	16 ± 6	NS
Booking SBP (mmHg; median ± IQR)	100 ± 10	105 ± 10	100 ± 10	0.04
Booking DBP (mmHg; median ± IQR)	60 ± 10	60 ± 10	60 ± 6.5	NS
Gestation at delivery (weeks; median ± IQR)	39 ± 2	39 ± 2	39 ± 2	0.79
Spontaneous or induced labour onset	188	58 (30.8%)	130 (69.1%)	<0.01
Vaginal delivery				
Emergency caesarean section	157	29 (18.5%)	128 (81.5%)	
No labour	31	29 (93.5%)	2 (6.5%)	
	41	33 (80.5%)	8 (19.5%)	<0.01
Duration of first stage (median ± IQR)	3:30 (± 3:37)	3:30 (± 3:30)	3:30 (± 3:30)	0.74
Duration of second stage (median ± IQR)	0:28 (± 1:34)	01:02 (± 1:45)	00:22 (± 1:27)	0.03
Intravenous syntocin, *n* (%)	86	34 (39.5%)	52 (60.5%)	0.96
Epidural, *n* (%)	70	39 (55.7%)	31 (44.3%)	<0.01

Intrapartum non-steroidal anti-inflammatory drugs, *n* (%)	45	41 (91.1%)	4 (88.9%)	<0.01
**Maternal outcomes**				
Instrumental delivery, *n* (%)<br>Forceps<br>Vacuum	34<br>6<br>28	9 (26.5%)<br>3 (50%)<br>6 (21.4%)	25 (73.5%)<br>3 (50%)<br>22 (78.6%)	0.89
Emergency caesarean section, *n* (%)	31	29 (93.5%)	2 (6.5%)	<0.01
Third- or fourth-degree perineal tear, *n* (%)	5	0 (0%)	5 (100%)	<0.01
Maternal ICU admission, *n* (%)	0	0 (0%)	0 (0%)	-
Length of stay following delivery (median ± IQR)	2 (± 2)	3 (± 1)	2 (± 1)	<0.01
Length of stay >3 days, *n* (%)	17	4 (23.5%)	13 (76.5%)	NS
Postpartum hypertension, *n* (%)<br>Required IV antihypertensive agents<br>Discharged with antihypertensive medications	19<br>0<br>4	6 (31.6%)<br>0 (0%)<br>4 (100%)	13 (68.4%)<br>0 (0%)<br>0 (0%)	0.02<br>-<br><0.01
Readmission due to hypertension, *n* (%)	3	0 (0%)	3 (100%)	NS
Maternal composite outcome*	5	2 (40%)	3 (60%)	NS
**Fetal outcomes**				
Male infant, *n* (%)	118	18 (15.3%)	100 (84.7%)	NS
1 minute APGAR score <9, *n* (%)	21	5 (23.8%)	16 (76.2%)	NS
5 minute APGAR score <9, *n* (%)	9	3 (33.3%)	6 (66.7%)	NS
Fetal weight (median ± IQR)	3468 (± 746)	3420 (± 904)	3473 (± 695)	NS

Small-for-gestational age at delivery (<10th centile)	13	3 (23.1%)	10 (76.9%)	NS
Intrauterine growth restriction at delivery (<5th centile)	5	1 (20%)	4 (80%)	NS
Preterm delivery (<37 weeks), *n* (%)	10	1 (10%)	9 (90%)	NS
Admission to special care nursery/neonatal intensive care unit, *n* (%)	35	8 (22.9%)	27 (77.1%)	NS
Fetal composite outcome^	40	9 (22.5%)	31 (77.5%)	NS

IH - intrapartum hypertension; SD – standard deviation; IQR – interquartile range; BMI – body mass index; SBP – systolic blood pressure; DBP – diastolic blood pressure; PET – preeclampsia; HTN – hypertension; NS – non-significant; SROM – spontaneous rupture of membranes; IOL – induction of labour; LSCS – lower segment caesarean section; GDM – gestational diabetes mellitus; PROM – preterm rupture of membranes; AFI – amniotic fluid index; APGAR - Appearance, Pulse, Grimace, Activity, and Respiration; ICU – intensive care unit; IV - intravenous

*Maternal composite outcome was defined as the composite of maternal mortality, stroke, acute coronary event, eclampsia, acute kidney injury, raised liver enzymes, pulmonary oedema, placental abruption, ICU admission or readmission within 6 weeks of delivery.

^Fetal composite outcome was defined as the composite of admission to the special care nursery or neonatal intensive care unit, stillbirth, preterm at delivery, neonatal mortality, neonatal seizures or intrauterine growth restriction (<5th centile).

Supplementary Table 2: Baseline demographics, intrapartum details and maternofetal outcomes for women with and without HDP

	Total (n=300)	With pre-existing HDP diagnosis (n = 18)	Without pre-existing HDP (n = 282)	p-value
Age (mean ± SD)	30.3 ± 5.2	33.3 ± 4.9	30.1 ± 5.2	0.02
Gravidity (median ± IQR)	2 ± 3	4 ± 3	2 ± 3	0.03
Parity (median ± IQR)	1 ± 2	2 ± 2	1 ± 2	<0.01
BMI (median ± IQR)	25.9 ± 9.0	29.1 ± 11.2	25.6 ± 8.9	NS
Gestation at booking (median ± IQR)	16 ± 6	16 ± 5	16 ± 6	NS
Booking SBP mmHg (median ± IQR)	102 ± 10	120 ± 2	100 ± 10	<0.01
Booking DBP mmHg (median ± IQR)	60 ± 10	75 ± 11	60 ± 8	<0.01
Gestation at delivery (median ± IQR)	39 ± 2	39 ± 2	39 ± 2	<0.01
At least first stage of labour<br>Normal vaginal delivery<br>Emergency caesarean section<br>No labour	249<br>216<br>33<br>51	8 (3.2%)<br>6 (2.8%)<br>2 (6.1%)<br>10 (19.6%)	241 (96.8%)<br>210 (97.2%)<br>31 (93.9%)<br>41 (80.4%)	<0.01
Duration of first stage (median ± IQR)	3:00 (± 3:15)	1:50 (± 3:50)	3:02 (± 3:15)	NS
Duration of second stage (median ± IQR)	00:19 (± 1:00)	00:13 (± 0:30)	00:20 (± 1:06)	NS
Intravenous syntocin, *n* (%)	91	4 (4.4%)	87 (95.6%)	NS
Epidural, *n* (%)	74	4 (5.4%)	70 (94.6%)	NS
Intrapartum non-steroidal anti-inflammatory drugs, *n* (%)	47	2 (11.1%)	45 (16%)	NS

**Maternal outcomes**				
Instrumental delivery, *n* (%)<br>Forceps<br>Vacuum	36<br>6<br>30	0 (0%)<br>0 (0%)<br>0 (0%)	36 (12.8%)<br>6 (2.1%)<br>30 (10.6%)	NS
Emergency caesarean section, *n* (%)	33	2 (6.5%)	31 (93.9%)	NS
Third- or fourth-degree perineal tear, *n* (%)	7	0 (0%)	7 (100%)	<0.01
Maternal ICU admission, *n* (%)	2	2 (100%)	0 (0%)	<0.01
Length of stay following delivery (median ± IQR)	2 (± 2)	3 (±2)	2 (± 1)	<0.01
Length of stay >3 days, *n* (%)	23	6 (26.1%)	17 (73.9%)	<0.01
Postpartum hypertension, *n* (%)<br>Required IV antihypertensive agents<br>Discharged with antihypertensive medications	36<br>3<br>17	15 (41.7%)<br>3 (100%)<br>13 (76.5%)	21 (58.3%)<br>0 (0%)<br>4 (23.5%)	<0.01<br><0.01<br><0.01
Readmission due to hypertension, *n* (%)	5	2 (40%)	3 (60%)	<0.01
Maternal composite outcome*	13	8 (61.5%)	5 (38.5%)	<0.01
**Fetal outcomes**				
Male infant, *n* (%)	152	10 (6.6%)	142 (93.4%)	NS
1 minute APGAR score <9, *n* (%)	27	3 (11.1%)	24 (88.9%)	NS
5 minute APGAR score <9, *n* (%)	15	3 (20%)	12 (80%)	0.02
Fetal weight (median ± IQR)	3445g (± 710)	3325 (± 871)	3445g (± 710)	NS
Small-for-gestational age at delivery (<10th centile)	16	1 (6.3%)	15 (93.8%)	NS

Intrauterine growth restriction at delivery (<5th centile)	7	1 (14.3%)	6 (85.7%)	NS
Preterm delivery (<37 weeks), *n* (%)	16	3 (18.8%)	13 (81.1%)	0.03
Admission to special care nursery/neonatal intensive care unit, *n* (%)	45	4 (8.9%)	41 (91.1%)	NS
Fetal composite outcome^	53	5 (9.4%)	48 (90.6%)	NS

IH - intrapartum hypertension; SD – standard deviation; IQR – interquartile range; BMI – body mass index; SBP – systolic blood pressure; DBP – diastolic blood pressure; PET – preeclampsia; HTN – hypertension; NS – non-significant; SROM – spontaneous rupture of membranes; IOL – induction of labour; LSCS – lower segment caesarean section; GDM – gestational diabetes mellitus; PROM – preterm rupture of membranes; AFI – amniotic fluid index; APGAR - Appearance, Pulse, Grimace, Activity, and Respiration; ICU – intensive care unit; IV - intravenous

*Maternal composite outcome was defined as the composite of maternal mortality, stroke, acute coronary event, eclampsia, acute kidney injury, raised liver enzymes, pulmonary oedema, placental abruption, ICU admission or readmission within 6 weeks of delivery.

^Fetal composite outcome was defined as the composite of admission to the special care nursery or neonatal intensive care unit, stillbirth, preterm at delivery, neonatal mortality, neonatal seizures or intrauterine growth restriction (<5th centile).
